# Adjusting heterogeneous ascertainment bias for genetic association analysis with extended families

**DOI:** 10.1186/s12881-015-0198-6

**Published:** 2015-08-19

**Authors:** Suyeon Park, Sungyoung Lee, Young Lee, Christine Herold, Basavaraj Hooli, Kristina Mullin, Taesung Park, Changsoon Park, Lars Bertram, Christoph Lange, Rudolph Tanzi, Sungho Won

**Affiliations:** Department of Applied Statistics, Chung-Ang University, Seoul, Korea; Center for Genome Science, National Institute of Health, Osong Health Technology Administration complex, Chungcheongbuk-do, Seoul Korea; Department of Biostatistics, Soonchunhyang University, College of Medicine, Seoul, Korea; Interdisciplinary Program in Bioinformatics, Seoul National University, Seoul, Korea; German Center for Neurodegenerative Diseases (DZNE), Sigmund-Freud-Str. 25, Bonn, 53127 Germany; Department of Biostatistics, Harvard School of Public Health, Boston, MA USA; Genetics and Aging Research Unit, MassGeneral Institute for Neurodegenerative Diseases, Department of Neurology, Massachusetts General Hospital, Harvard Medical School, Massachusetts, USA; Department of Statistics, Seoul National University, Seoul, Korea; Department of Vertebrate Genomics, Neuropsychiatric Genetics Group, Max Planck Institute for Molecular Genetics, Berlin, Germany; Department of Medicine, School of Public Health, Imperial College London, London, UK; Harvard Medical School, Boston, MA USA; Institute for Genomic Mathematics, University of Bonn, Bonn, Germany; German Center for Neurodegenerative Diseases, Bonn, Germany; Department of Public Health Science, Seoul National University, Seoul, Korea; Institute of Health and Environment, Seoul National University, Seoul, Korea; National Cancer Center, Seoul, Korea

**Keywords:** Family-based association analysis, Ascertainment, Liability model

## Abstract

**Background:**

In family-based association analysis, each family is typically ascertained from a single proband, which renders the effects of ascertainment bias heterogeneous among family members. This is contrary to case–control studies, and may introduce sample or ascertainment bias. Statistical efficiency is affected by ascertainment bias, and careful adjustment can lead to substantial improvements in statistical power. However, genetic association analysis has often been conducted using family-based designs, without addressing the fact that each proband in a family has had a great influence on the probability for each family member to be affected.

**Method:**

We propose a powerful and efficient statistic for genetic association analysis that considered the heterogeneity of ascertainment bias among family members, under the assumption that both prevalence and heritability of disease are available. With extensive simulation studies, we showed that the proposed method performed better than the existing methods, particularly for diseases with large heritability.

**Results:**

We applied the proposed method to the genome-wide association analysis of Alzheimer’s disease. Four significant associations with the proposed method were found.

**Conclusion:**

Our significant findings illustrated the practical importance of this new analysis method.

**Electronic supplementary material:**

The online version of this article (doi:10.1186/s12881-015-0198-6) contains supplementary material, which is available to authorized users.

## Background

Genome-wide association studies (GWASs) have been used to identify many genes involved in human diseases, and during the last decade, many disease-susceptibility variants have been identified. However, despite these successes, we have found that variants discovered from GWASs often explain only a small proportion of the heritability of diseases [1, 2]. For example, SNPs significantly associated with human height explain only about 5 % of phenotypic variance, despite studies of tens of thousands of people [3]. Many reasons, such as rare causal variants and gene/gene interactions, have been attributed to this so-called “missing heritability”. However, the low power induced by the multiple-testing problem is still an intractable issue in GWASs, and further investigations of the most efficient strategies for genetic association analysis are necessary.

Careful selection of samples based on phenotypes can lead to improved power for the discovery of risk variants [4–11]. One such example is the extreme discordant sib-pair design in linkage analysis, which may result in a substantial increase in statistical power when compared to other sib-pair designs [11, 12]. Similarly, ascertaining the extremes of quantitative phenotypes from large population cohorts has also been shown to increase the power to identify associated variants [13–15]. In such a design, the effect of ascertainment conditions are homogeneous between individuals, and existing methods, such as the Cochran-Armitage(CA) trend test [16], can be an efficient choice. However, in association analysis using extended families, the effects of ascertainment bias are often heterogeneous among family members, and depending on their relationships with probands, different magnitudes of ascertainment bias may be generated. In particular, the probability of each individual being affected when his or her relatives are affected is similar to the prevalence, if the heritability is small, which indicates that the heterogeneous effect of the ascertainment bias depends on the magnitude of heritability. However, the heterogeneous effects of ascertainment conditions and the influence of heritability on it have not yet been investigated, and should therefore be taken into account for association analysis.

Recently, the CA trend test was extended for association analysis of dichotomous phenotypes with family-based samples [17, 18]. These statistics compares the genotype frequencies between affected and unaffected individuals, and the genetic association with family-based samples is tested by building a genotype correlation matrix with either kinship coefficients or an empirical correlation matrix estimated from large-scale genetic data. This approach has been extended to include family members with known phenotypes and missing genotypes or *vice versa*. By the nature of these statistics, it performs well for ascertained family-based samples and it can be an efficient choice, even for a case–control design, if the relatives’ phenotype information is available. However, their statistical efficiency is affected by the heterogeneous effect of the ascertainment bias on family members, and for extended families, its effects on statistical efficiency can be substantial.

In this report, we consider the heterogeneous effects of the ascertainment bias on family members for dichotomous phenotypes. By the nature of the proposed methods, individuals with missing genotypes and non-missing phenotypes can be utilized, and incorporation of the estimated kinship matrix to the proposed statistic provided robustness against the population substructure. The proposed method consists of two steps; the probability for each family member to be affected was calculated using a latent continuous liability [19], and then this probability is incorporated into a quasi-likelihood score test. With an extensive simulation, we showed that the proposed method performed better than the existing methods, particularly for a disease with large heritability. Application of our method to Alzheimer’s disease (AD) demonstrated its practical use in the detection of genetic associations in ascertained family-based samples.

## Methods

### Notations and statistic

**Fig. 1:**
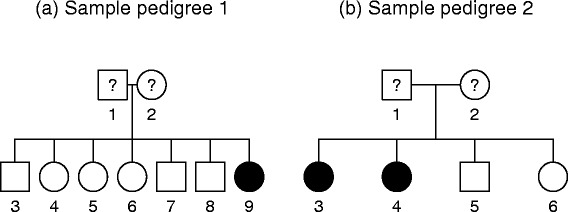
Two sample pedigree structures from AD data; (**a**) individual 9 of family 1 was selected as a "proband" and individual 3–8 of family 1 were selcted as "non-probands" (**b**) individual 3 of family 2 was selected as a "proband" and individual 4–6 of family 2 were selcted as "non-probands"

We assumed that there were *n* families and *n*_*i*_ family members in each family. We considered the situation where the family of size *n*_*i*_ was ascertained because it contained a particular set of *p*_*i*_ members, and we let *q*_*i*_ = *n*_*i*_ – *p*_*i*_. We called the members of the set of *p*_*i*_ family members “probands”, and the remaining *q*_*i*_ individuals “non-probands”. To provide a clearer motivation on this concept, we randomly selected two families, family 1 and 2, from our AD data (see Fig. 1). In family 1 (Fig. 1-(a)), individual 9 was diagnosed as AD and individuals 3–8 were selected as her relatives for genetic analysis. In family 2 (Fig. 1-(b)), individual 3 was diagnosed as AD, and individuals 4–6 were selected. Therefore *p*_1_ = *p*_2_ = 1, *q*_1_ = 6 and *q*_2_ = 3 in this example. In real data analysis, *p*_*i*_ is often 1 and *q*_*i*_ = *n*_*i*_ – 1. We assumed that *N* individuals were available and thus *N* = ∑_*i*_*n*_*i*_. The genotypes were coded as 0, 1, or 2, according to the number of disease alleles. *x*_*ij*_^*P*^ and *x*_*i* ' *j* '_^*N*^ were defined as the genotypes of proband *j* and non-proband *j'* in family *i* and family *i'*, respectively. Phenotypes were coded as 0 for an unaffected individual and 1 for an affected individual. If we let the prevalence of the disease be *p*, a missing phenotype was coded as *p*. We denoted the phenotypes of a proband and non-proband by *y*_*ij*_^*P*^ and *y*_*i* ' *j* '_^*N*^, respectively, and the vectors for genotypes and phenotypes in family *i* were defined by

$$ {\mathbf{X}}_i^P=\left(\kern1em \begin{array}{c}{x}_{i1}^P\kern1em \\ {}\kern1em {x}_{i2}^P\kern1em \\ {}\kern1em \vdots \kern1em \\ {}\kern1em {x}_{i{p}_i}^P\end{array}\kern1em \right),\kern0.5em {\mathbf{X}}_i^N=\left(\kern1em \begin{array}{c}{x}_{i1}^N\kern1em \\ {}\kern1em {x}_{i2}^N\kern1em \\ {}\kern1em \vdots \kern1em \\ {}\kern1em {x}_{i{q}_i}^N\end{array}\kern1em \right)\kern0.5em ,\kern0.5em {\mathbf{X}}_i=\left(\kern1em \begin{array}{c}{\mathbf{X}}_i^P\kern1em \\ {}\kern1em {\mathbf{X}}_i^N\end{array}\kern1em \right),\kern0.5em {\mathbf{Y}}_i^P=\left(\kern1em \begin{array}{c}{y}_{i1}^P\kern1em \\ {}\kern1em {y}_{i2}^P\kern1em \\ {}\kern1em \vdots \kern1em \\ {}\kern1em {y}_{i{p}_i}^P\end{array}\kern1em \right),\kern0.5em {\mathbf{Y}}_i^N=\left(\kern1em \begin{array}{c}{y}_{i1}^N\kern1em \\ {}\kern1em {y}_{i2}^N\kern1em \\ {}\kern1em \vdots \kern1em \\ {}\kern1em {y}_{i{q}_i}^N\end{array}\kern1em \right),\kern0.5em \mathrm{and}\kern0.5em {\mathbf{Y}}_i=\left(\kern1em \begin{array}{c}{\mathbf{Y}}_i^P\kern1em \\ {}\kern1em {\mathbf{Y}}_i^N\end{array}\kern1em \right) $$

We also denoted the *w* × *w* identity matrix by **I**_*w*_, and the *w* × 1 column vector **1**_*w*_ indicated a vector in which all elements were 1. Let *π*_*ijj* '_^*P*^ and *π*_*ijj* '_^*N*^ be the kinship coefficient between probands *j* and *j'* in family *i*, and non-proband *j* and *j'* in family *i*, respectively*.* In addition, we let *π*_*ijj* '_^*PN*^ be the kinship coefficient between proband *j* and non-proband *j'* in family *i*, and let *d*_*ij*_^*P*^ and *d*_*ij'*_^*N*^ be the inbreeding coefficient for proband *j* and non-proband *j'* in family *i*, respectively*.* The inbreeding coefficient is the parameter that quantifies the departure from Hardy-Weinberg equilibrium (HWE) and ranges from 0 to 1. Several approaches [20, 21] that can estimate *d*_*ij*_ have been proposed. We let

$$ {\mathbf{R}}_i^P=\left(\kern1em \begin{array}{ccc}1+{d}_{i1}^P\kern1em & \kern1em \cdots \kern1em & \kern1em 2{\pi}_{i1{p}_i}^P\kern1em \\ {}\kern1em \vdots \kern1em & \kern1em \ddots \kern1em & \kern1em \vdots \kern1em \\ {}\kern1em 2{\pi}_{i{p}_i1}^P\kern1em & \kern1em \dots \kern1em & \kern1em 1+{d}_{i{p}_i}^P\end{array}\kern1em \right),\kern0.5em {\mathbf{R}}_i^N=\left(\kern1em \begin{array}{ccc}1+{d}_{i1}^N\kern1em & \kern1em \cdots \kern1em & \kern1em 2{\pi}_{i1{q}_i}^N\kern1em \\ {}\kern1em \vdots \kern1em & \kern1em \ddots \kern1em & \kern1em \vdots \kern1em \\ {}\kern1em 2{\pi}_{i{q}_i1}^N\kern1em & \kern1em \dots \kern1em & \kern1em 1+{d}_{i{q}_i}^N\end{array}\kern1em \right),\kern0.5em {\mathbf{R}}_i^{PN}=\left(\kern1em \begin{array}{ccc}2{\pi}_{i11}^{PN}\kern1em & \kern1em \cdots \kern1em & \kern1em 2{\pi}_{i1{q}_i}^{PN}\kern1em \\ {}\kern1em \vdots \kern1em & \kern1em \ddots \kern1em & \kern1em \vdots \kern1em \\ {}\kern1em 2{\pi}_{i{p}_i1}^{PN}\kern1em & \kern1em \dots \kern1em & \kern1em 2{\pi}_{i{p}_i{q}_i}^{PN}\end{array}\kern1em \right), $$

and **R**_*i*_ is defined by

$$ {\mathbf{R}}_i=\left(\kern1em \begin{array}{c}{\mathbf{R}}_i^P\kern1em \\ {}\kern1em {\left({\mathbf{R}}_i^{PN}\right)}^t\kern1em \end{array}\kern0.5em \begin{array}{c}\kern1em {\mathbf{R}}_i^{PN}\kern1em \\ {}\kern1em {\mathbf{R}}_i^N\end{array}\kern1em \right) $$

If we let *q*_*A*_ be the disease allele frequency, *E*(**X**_*i*_) was $2{q}_A{1}_{n_i}$, and *q*_*A*_ is estimated with the best linear unbiased estimator (BLUE). var(**X**_*i*_) is expressed by *σ*^2^**R**_*i*_, and *σ*^2^ is equal to 2*q*_*A*_(1 –*q*_*A*_) under HWE.

When we analyzes the distribution of genotypes as in the FBAT approach, the statistical efficiency of the test statistic could be improved by adjustments of the phenotype with the so-called offset [22]. If we let *μ*_*ij*_^*P*^ and *μ*_*i'j'*_^*N*^ be offsets for proband *j* and non-proband *j'* in family *i* and family *i'*, respectively, the offset vector for family *i* is defined as

$$ {\upmu}_i^P=\left(\kern1em \begin{array}{c}{\mu}_{i1}^P\kern1em \\ {}\kern1em {\mu}_{i2}^P\kern1em \\ {}\kern1em \vdots \kern1em \\ {}\kern1em {\mu}_{i{p}_i}^P\end{array}\kern1em \right),\kern0.5em {\upmu}_i^N=\left(\kern1em \begin{array}{c}{\mu}_{i1}^N\kern1em \\ {}\kern1em {\mu}_{i2}^N\kern1em \\ {}\kern1em \vdots \kern1em \\ {}\kern1em {\mu}_{i{q}_i}^N\end{array}\kern1em \right),\kern0.5em \mathrm{and}\kern0.5em {\upmu}_i=\left(\kern1em \begin{array}{c}{\upmu}_i^P\kern1em \\ {}\kern1em {\upmu}_i^N\end{array}\kern1em \right) $$

Setting **T**_***i***_ = **Y**_*i*_–**μ**_*i*_, we can define

$$ \mathbf{X}=\left(\kern1em \begin{array}{c}{\mathbf{X}}_1\kern1em \\ {}\kern1em {\mathbf{X}}_2\kern1em \\ {}\kern1em \vdots \end{array}\kern1em \right),\kern0.5em \mathbf{Y}=\left(\kern1em \begin{array}{c}{\mathbf{Y}}_1\kern1em \\ {}\kern1em {\mathbf{Y}}_2\kern1em \\ {}\kern1em \vdots \end{array}\kern1em \right),\kern0.5em \mathbf{T}=\left(\kern1em \begin{array}{c}{\mathbf{T}}_1\kern1em \\ {}\kern1em {\mathbf{T}}_2\kern1em \\ {}\kern1em \vdots \end{array}\kern1em \right),\kern0.5em \mathrm{and}\kern0.5em \mathbf{R}=\left(\kern1em \begin{array}{c}{\mathbf{R}}_1\kern1em \\ {}\kern1em 0\kern1em \\ {}\kern1em \vdots \kern1em \end{array}\begin{array}{c}\kern1em 0\kern1em \\ {}\kern1em {\mathbf{R}}_2\kern1em \\ {}\kern1em \vdots \kern1em \end{array}\begin{array}{c}\kern1em \cdots \kern1em \\ {}\kern1em \cdots \kern1em \\ {}\kern1em \ddots \end{array}\kern1em \right). $$

We denoted a minor allele frequency (MAF) of a variant in unaffected individuals by *q*. We assumed [18] that for a constant γ,

$$ E\left(\mathbf{X}\Big|\mathbf{T}\right)=2p{\mathbf{1}}_N+\gamma \mathbf{T}, $$

where 0 < 2*p* + *γ* < 1. Then, the score for a variant [18, 23] can be defined by

$$ S={\mathbf{T}}^t\left(\mathbf{X}-\widehat{E}\left(\mathbf{X}\right)\right)\kern0.5em \mathrm{and}\kern0.5em \widehat{E}\left(\mathbf{X}\right)={\mathbf{1}}_N{\left({\mathbf{1}}_N^t{\mathbf{R}}^{-1}{\mathbf{1}}_N\right)}^{-1}{\mathbf{1}}_N^t{\mathbf{R}}^{-1}\mathbf{X}. $$

The variance of *S* is

$$ var(S)={\sigma}^2{\mathbf{T}}^t{\mathbf{V}}^{-1}\left(\mathbf{R}-{\mathbf{1}}_N{\left({\mathbf{1}}_N^t{\mathbf{R}}^{-1}{\mathbf{1}}_N\right)}^{-1}{\mathbf{1}}_N^t\right){\mathbf{V}}^{-1}\mathbf{T}, $$

and we considered the following statistic [17, 18]:

$$ \frac{{\mathbf{T}}^t\left({\mathbf{I}}_N-{\left({\mathbf{1}}_N^t{\mathbf{R}}^{-1}{\mathbf{1}}_N\right)}^{-1}{\mathbf{1}}_N^t{\mathbf{R}}^{-1}\right)\mathbf{X}}{\sqrt{\sigma^2{\mathbf{T}}^t\left(\mathbf{R}-{\mathbf{1}}_N{\left({\mathbf{1}}_N^t{\mathbf{R}}^{-1}{\mathbf{1}}_N\right)}^{-1}{\mathbf{1}}_N^t\right)\mathbf{T}}}\sim N\left(0,1\right)\mathrm{if}\gamma =0. $$

This statistic will be denoted by *WL* in the remainder of this report.

### Adjusting the heterogeneous ascertainment bias

Families are often selected based on some probands, and the probability for family members to be affected depends on their relationship with the probands. Additional file 1 shows that the incorporation of conditional probability of each individual being affected to *WL* as offset lead to asymptotically smaller variance and therefore the adjustment of heterogeneous ascertainment bias is required to improve the statistical power of *WL*. This probability could be estimated with the liability model if the heritabilities, *h*^*2*^, and prevalence, *p*, were available. We let *l*_*ij*_^*P*^ and *l*_*i* ' *j* '_^*N*^ be the liability of proband *j* and non-proband *j'* in family *i* and family *i'*, respectively, and let ${\mathbf{L}}_i^P=\left({l}_{i1}^P\kern0.5em ,\dots, \kern0.5em {l}_{i{p}_i}^P\right)$ and ${\mathbf{L}}_i^N=\left({l}_{i1}^N\kern0.5em ,\dots, \kern0.5em {l}_{i{q}_i}^N\right)$. We assumed that each liability followed the standard normal distribution, and their joint distributions were

$$ \left(\kern0.75em \begin{array}{c}{L}_i^P\kern0.75em \\ {}\kern0.5em {L}_i^N\end{array}\kern0.75em \right)\sim MVN\left(0,{h}^2\left(\kern0.75em \begin{array}{c}{\mathbf{R}}_i^P\kern1em \\ {}\kern0.75em {\left({\mathbf{R}}_i^{PN}\right)}^t\kern0.62em \end{array}\begin{array}{c}\kern1em {\mathbf{R}}_i^{PN}\kern1em \\ {}\kern1em {\mathbf{R}}_i^N\end{array}\right)\kern1em +\left(1-{h}^2\right){\mathbf{I}}_{n_i}\right). $$

Benchek and Morris [24] reported that significant asymptotic biases are likely to arise when the multivariate normal (MVN) liability assumption is not met and in such a case, different assumptions should be considered. We assume that **M**_*i*_^*P* *^ and **V**_*i*_^*P* *^ are the expectation and variances of *L*_*i*_^*P*^ when their disease statuses are conditioned. If all probands are affected, they becomes

$$ {{\mathbf{M}}_i}^{P^{\ast }}\equiv E\left({L_i}^P\Big|{l_{i1}}^P>c,\dots, {l_{i{p}_i}}^P>c\right) $$

and

$$ {{\mathbf{V}}_i}^{P^{\ast }}\equiv var\left({L_i}^P\Big|{l_{i1}}^P>c,\dots, {l_{i{p}_i}}^P>c\right). $$

They can be calculated with the numerical algorithms [25]. If *p*_*i*_ is 1, both can be simply calculated. We denote the cumulative and probability density function of standard normal distribution by Ф(·) and ϕ(·). If we let *c* be the (1–*p*)th quantile of the standard normal distribution, **M**_*i*_^*P* *^ and **V**_*i*_^*P* *^ becomes

$$ {\mathbf{M}}_i^{P\ast}\Big\{\kern1em \begin{array}{c}\phi (c)/\left[1-\varPhi (x)\right]\kern1em \\ {}\kern1em -\phi (c)/\varPhi (x)\kern1em \end{array}\kern2em \begin{array}{c}\kern1em \mathrm{if}\kern0.5em {y}_{i1}^P=1\kern1em \\ {}\kern1em \mathrm{if}\kern0.5em {y}_{i1}^P=0\kern1em \end{array},\kern1em \mathrm{and}\kern0.5em {\mathbf{V}}_i^{P\ast}\kern0.5em =1-{\left({\mathbf{M}}_i^{P\ast}\right)}^2+{\mathbf{M}}_i^{P\ast }c. $$

With Pearson-Aitken formula [26, 27], we could obtain the conditional mean and variance-covariance matrix of **L**_*i*_^*N*^ given ${\mathbf{L}}_i^P>{1}_{p_i}\kern0.5em \cdot c$ as follows:

$$ {\mathbf{M}}_i^{N\ast }=0+{\left({\mathbf{R}}_i^{PN}\right)}^t{\left({\mathbf{V}}_i^P\right)}^{-1}\left({\mathbf{M}}_i^{P\ast }-0\right) $$

and

$$ {\mathbf{V}}_i^{N\ast }={\mathbf{V}}_i^N+{\left({\mathbf{R}}_i^{PN}\right)}^t\left({\left({\mathbf{V}}_i^P\right)}^{-1}-{\left({\mathbf{V}}_i^P\right)}^{-1}{\mathbf{V}}_i^{P\ast }{\left({\mathbf{V}}_i^P\right)}^{-1}\right){\mathbf{R}}_i^{PN}. $$

We denoted the *j*th element in **M**_*i*_^*N* *^ by *m*_*j*_^*N* *^ and the *j*th diagonal element in **V**_*i*_^*N* *^ by *v*_*j*_^*N* *^. Then the probability of being affected for a non-proband under multivariate normality of the liabilities could be calculated as

$$ \varPhi \left(\frac{c-{m}_j^{N\ast }}{v_j^{N\ast }}\right), $$

and this will be incorporated into the proposed statistic as offset. Thus far, we have assumed that there was a well-designed set of *p*_*i*_ individuals who were “probands”, and for this situation, we calculated the statistic as indicated and denoted *FQLS*_1_. However in practice, different ascertainment condition such as sequential sampling frame [28] are often utilized, and the set of *p*_*i*_ individuals will not be well defined. For this situation, we calculated the probability for each individual to be affected under the assumption that all the other family members were “probands”, and thus *p*_*i*_ = *n*_*i*_ – 1 and *q*_*i*_ = 1. The statistic calculated this way was denoted by *FQLS*_2_.

## Results

### The simulation model

**Fig. 2:**
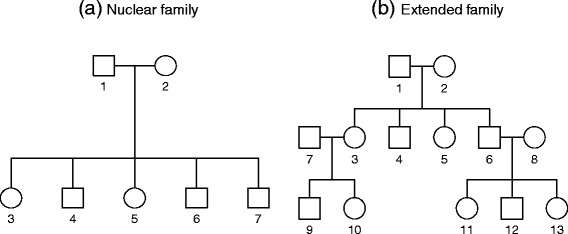
Family tree. There are two different types of family structures, including **(a)** nuclear family and **(b)** extended family, which were considered in our simulation study

In our simulation studies, we considered two types of family structures; nuclear families with five offspring and the extended families that consist of 13 individuals along 3 generations (see Fig. 2). The latter will be called extended families in the remainder of this report. The disease allele frequency, *p*, was assumed to be 0.2. If we denoted the disease allele frequency by *q*_*A*_, the genotype frequencies for *AA*, *Aa*, and *aa* became *q*_*A*_^2^, 2*q*_*A*_(1 – *q*_*A*_), and (1 – *q*_*A*_)^2^ under HWE, respectively, and founders’ genotypes were generated under the corresponding multinomial distribution. The genotypes for non-founders were generated with randomly generated Mendelian transmission. The disease status was generated with the liability threshold model. Once continuous liabilities that consisted of polygenic effects and random errors were generated, they were transformed to being affected if they were larger than the threshold; and otherwise, they were considered to be unaffected. The threshold was chosen to preserve the prevalence, and prevalence was assumed to be 0.2. Continuous liability was determined by combining the phenotypic mean, polygenic effect, main genetic effect, and random error. The main genetic effect for each individual was the product of *β* and the number of disease alleles. If we denoted the relative proportion of the phenotypic variance attributable to the main disease gene by *h*_*a*_^2^, and *h*^2^ was a heritability for continuous liability, *β* was calculated by

$$ \beta =\sqrt{\frac{h_a^2}{2{q}_A\left(1-{q}_A\right)\left(1-{h}^2\right)}}. $$

For the evaluation of type-1 errors and power, *h*_*a*_^2^ was assumed to be 0 and 0.005, respectively. Phenotypic correlations between family-members were explained by the polygenic effects. Parental polygenic effects were generated from *N*(0, *h*^2^), and *h*^2^ was assumed to be 0.2, 0.5, or 0.8. For non-founders, the average of maternal and paternal polygenic effects was combined with the values independently sampled from *N*(0, 0.5 *h*^2^) for the polygenic effects of offspring. Random errors were generated from *N*(0, *σ*_*e*_^2^ = 1–*h*^2^). For each replicate, sampling was repeated until a given number of ascertained families was generated. Type-1 error estimates were calculated with 5000 replicates, and empirical power estimates were calculated with 1000 replicates.

### Evaluation of the proposed methods with simulated data

**Fig. 3:**
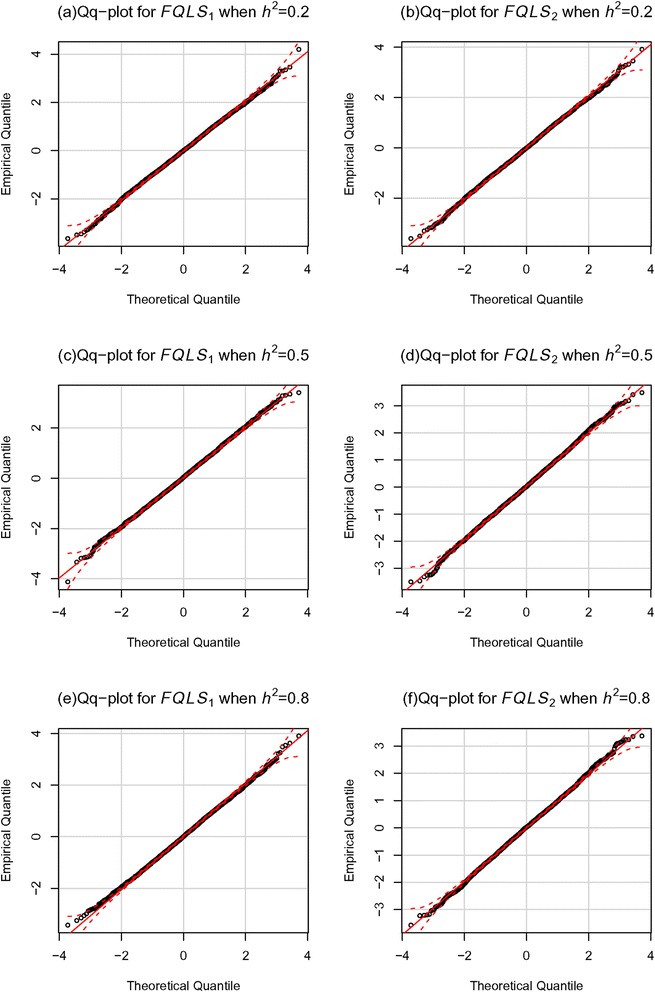
QQ plots for *FQLS*_1_ and *FQLS*_2_ under the null hypothesis. QQplots for *FQLS*_1_ and *FQLS*_2_ are obtaind when h2 is 0.2((**a**), (**b**)), 0.5((**c**), (**d**)), or 0.8((**e**), (**f**)). P-values were calculated based on 5000 replicates when the number of families was 900. The genetic effect β was assumed to be 0, and the minor allele frequency was 0.2

The empirical type-1 errors for *FQLS*_1_ and *FQLS*_2_ were evaluated from 5000 replicates under the situation of no association (*h*_*a*_^2^ = 0), and 900 nuclear families with five offspring in Fig. 2 were generated for each replicate. Fig. 3 shows the quantile quantile (QQ) plots from 5000 replicates, and the nominal significance levels for both methods were preserved for various significance levels. We also estimated the empirical type-1 error rates at the 0.01 and 0.05 significance levels; the empirical type-1 error estimates of *FQLS*_1_ and *FQLS*_2_ preserved these nominal significance levels (Table 1). These results verified that the use of the approximation to the standard normal distribution resulted in an accurate assessment of significance for the proposed methods.

**Table 1:** Empirical type-1 error estimates. The empirical type-1 error rates and their 95 % confidence intervals were estimated with 5000 replicates at the 0.01 and 0.05 significance level for *h* ^2^ = 0.2, 0.5, and 0.8. The number of families was assumed to be 900, and the disease allele frequency was 0.2

	*h* ^2^	Statistics	Type-1 error estimates	95 % confidence interval	
				Lower	Upper
0.01	0.2	*FQLS*_1_	0.011	0.008	0.014
		*FQLS*_2_	0.011	0.008	0.013
	0.5	*FQLS*_1_	0.010	0.007	0.013
		*FQLS*_2_	0.009	0.007	0.012
	0.8	*FQLS*_1_	0.009	0.006	0.011
		*FQLS*_2_	0.009	0.007	0.012
0.05	0.2	*FQLS*_1_	0.049	0.043	0.055
		*FQLS*_2_	0.049	0.043	0.055
	0.5	*FQLS*_1_	0.050	0.044	0.056
		*FQLS*_2_	0.053	0.047	0.059
	0.8	*FQLS*_1_	0.047	0.042	0.053
		*FQLS*_2_	0.053	0.047	0.059

The empirical powers at the various significance levels were measured based on 1000 replicates at the 0.01 and 0.001 significance levels. The relative proportion, *h*_*a*_^2^, of phenotypic variance attributable to the main disease gene, 2*p*_*A*_(1 – *p*_*A*_)*β*^2^, was assumed to be 0.005, and nuclear and extended families in Fig. 2 were considered for the power comparison. In the first simulation setting, the numbers of nuclear families were assumed to be 100, 300, 600, 900, 1200, and 1400, and half of the families were ascertained if the number of affected family members was larger than or equal to *n*_*proband*_, and the other half of the families were ascertained if the number of unaffected family members was larger than or equal to *n*_*proband*_. Therefore, if 100 nuclear families were generated, half of nuclear families should have more than or equal to *n*_*proband*_ affected family members, and the other half should have at least *n*_*proband*_ unaffected family members. We assumed that the heritabilities were 0.2, 0.5, and 0.8, and results are shown in Tables 2, 3, 4, respectively. In the second simulation setting, the numbers of extended families were assumed to be 100, 300, 600, and 900, and all families were ascertained if the number of affected amily members was larger than or equal to *n*_*proband*_. Empirical power estimates for scenario 2 were calculated when *h*^2^ = 0.2, 0.5, and 0.8, and the data are shown in Tables 5, 6, 7, respectively. Our results showed that either *FQLS*_1_ or *FQLS*_2_ was usually the most efficient statistic, and the least efficiency was provided from *WL*. In particular, the power gap between the proposed methods and *WL* was largest if *h*^2^ was 0.8, which indicates that power improvement may be proportional to the heritability. If *h*^2^ was 0.2, the proposed methods were only slightly better than *WL*. While all methods in our power comparison focused on the distribution of genotypes to calculate statistics, the proposed methods uniquely considered the heterogeneous effects of ascertainment bias among family members which were proportional to the magnitude of heritability; this explained the power improvement of the proposed methods. Furthermore the differences of empirical power estimates from *WL* and the proposed methods are larger for Tables 5, 6, 7 than Tables 2, 3, 4, which indicates that the heterogeneity of ascertainment condition may be positively related with family size and the proposed methods become more efficient for large families. Last our simulation results show that *FQLS*_2_ was slightly better than *FQLS*_1_, and this may be induced by the uncertainty of probands in our simulation studies. Therefore, we concluded that the incorporation of a sampling scheme to the offset could make a substantial difference, and test statistic should be carefully selected depending on type of sampling scheme.

**Table 2:** Empirical power estimates for scenario 1 when *h* ^2^ is 0.2. The empirical power estimates for scenario 1 were calculated with 1000 replicates at the both 0.01 and 0.001 significance levels. The disease allele frequency was assumed to be 0.2, and the prevalence was assumed to 0.2. The relative phenotypic variance attributable to the main disease gene was assumed to be 0.005

	*n*_*proband*_	Statistic	*N*					
			100	300	600	900	1200	1400
0.01	1	*WL*	0.027	0.062	0.129	0.219	0.293	0.369
		*FQLS*_1_	**0.029**	**0.064**	0.122	0.220	0.304	0.372
		*FQLS*_2_	**0.029**	0.059	**0.129**	**0.230**	**0.306**	**0.385**
	2	*WL*	0.033	**0.076**	0.165	0.295	0.415	0.456
		*FQLS*_1_	**0.036**	0.073	0.166	0.309	0.418	0.461
		*FQLS*_2_	0.035	**0.076**	**0.178**	**0.312**	**0.422**	**0.465**
	3	*WL*	**0.040**	0.103	**0.257**	0.398	0.519	0.609
		*FQLS*_1_	0.039	**0.112**	**0.257**	**0.404**	0.526	**0.623**
		*FQLS*_2_	0.036	0.110	0.253	0.407	**0.527**	0.621
	4	*WL*	0.041	0.127	0.297	0.497	0.626	0.720
		*FQLS*_1_	0.044	**0.129**	**0.307**	**0.502**	0.626	0.719
		*FQLS*_2_	**0.046**	0.126	**0.307**	0.493	**0.637**	**0.726**
0.001	1	*WL*	**0.005**	**0.012**	**0.038**	0.075	0.113	0.153
		*FQLS*_1_	**0.005**	**0.012**	**0.038**	**0.076**	**0.115**	0.157
		*FQLS*_2_	0.003	0.010	**0.038**	**0.076**	0.106	**0.163**
	2	*WL*	0.006	**0.019**	0.060	0.098	0.193	0.224
		*FQLS*_1_	**0.007**	0.018	0.059	0.099	**0.199**	0.217
		*FQLS*_2_	0.006	**0.019**	**0.064**	**0.100**	0.197	**0.219**
	3	*WL*	0.004	0.018	0.086	0.164	0.267	0.337
		*FQLS*_1_	0.007	0.020	**0.091**	0.162	**0.275**	0.333
		*FQLS*_2_	**0.008**	**0.023**	0.087	**0.165**	**0.275**	**0.348**
	4	*WL*	**0.010**	0.029	0.116	0.231	0.309	0.451
		*FQLS*_1_	0.009	0.029	0.116	0.228	**0.370**	0.449
		*FQLS*_2_	0.009	**0.031**	**0.118**	**0.233**	0.363	**0.459**

The bold text indicates the highest empirical estimate of the power for each situation

The bold text indicates the highest empirical estimate of the power for each situation

**Table 3:** Empirical power estimates for scenario 1 when *h* ^2^ is 0.5. The empirical power estimates for scenario 1 were calculated with 1000 replicates at the both 0.1 and 0.001 significance levels. The disease allele frequency was assumed to be 0.2, and the prevalence was assumed to 0.2. The relative phenotypic variance attributable to the main disease gene was assumed to be 0.005

	*n*_*proband*_	Statistic	*N*					
			100	300	600	900	1200	1400
0.01	1	*WL*	0.052	0.142	0.369	0.518	0.682	0.765
		*FQLS*_1_	0.053	0.145	0.352	0.523	0.681	0.766
		*FQLS*_2_	**0.056**	**0.151**	**0.389**	**0.543**	**0.702**	**0.796**
	2	*WL*	0.053	0.174	0.396	0.616	0.761	0.829
		*FQLS*_1_	**0.054**	0.183	0.400	0.619	0.780	0.834
		*FQLS*_2_	0.053	**0.202**	**0.422**	**0.658**	**0.799**	**0.859**
	3	*WL*	0.068	0.118	0.470	0.692	0.808	0.818
		*FQLS*_1_	**0.073**	0.216	0.489	0.705	**0.826**	**0.905**
		*FQLS*_2_	0.067	**0.232**	**0.498**	**0.729**	**0.826**	0.904
	4	*WL*	0.066	0.222	0.528	0.755	0.860	0.938
		*FQLS*_1_	0.068	**0.250**	**0.560**	**0.774**	**0.882**	**0.939**
		*FQLS*_2_	**0.072**	0.244	0.544	0.766	0.870	0.935
0.001	1	*WL*	0.012	0.034	0.143	0.247	0.376	0.505
		*FQLS*_1_	**0.015**	**0.046**	0.142	0.245	0.387	0.496
		*FQLS*_2_	0.013	**0.046**	**0.169**	**0.305**	**0.442**	**0.567**
	2	*WL*	0.008	0.060	0.151	0.342	0.494	0.612
		*FQLS*_1_	**0.014**	**0.055**	0.163	0.357	0.512	0.640
		*FQLS*_2_	0.012	**0.055**	**0.184**	**0.378**	**0.538**	**0.648**
	3	*WL*	0.005	0.033	0.223	0.404	0.579	0.595
		*FQLS*_1_	0.008	0.076	0.235	0.432	0.604	**0.717**
		*FQLS*_2_	**0.010**	**0.078**	**0.236**	**0.438**	**0.610**	0.699
	4	*WL*	0.010	0.079	0.274	0.484	0.655	0.763
		*FQLS*_1_	0.008	**0.088**	**0.296**	**0.490**	**0.677**	**0.783**
		*FQLS*_2_	**0.014**	0.084	0.280	0.474	0.671	0.769

The bold text indicates the highest empirical estimate of the power for each situation

The bold text indicates the highest empirical estimate of the power for each situation

**Table 4:** Empirical power estimates for scenario 1 when *h* ^2^ is 0.8. The empirical power estimates for scenario 1 were calculated with 1000 replicates at the both 0.01 and 0.001 significance levels. The disease allele frequency was assumed to be 0.2, and the prevalence was assumed to 0.2. The relative phenotypic variance attributable to the main disease gene was assumed to be 0.005

	*n*_*proband*_	Statistic	*N*					
			100	300	600	900	1200	1400
0.01	1	*WL*	0.068	0.233	0.470	0.699	0.819	0.903
		*FQLS*_1_	0.071	0.238	0.471	0.717	0.841	0.896
		*FQLS*_2_	**0.078**	**0.298**	**0.559**	**0.817**	**0.899**	**0.943**
	2	*WL*	0.071	0.222	0.521	0.708	0.881	0.937
		*FQLS*_1_	0.080	0.304	0.568	0.788	0.907	0.942
		*FQLS*_2_	**0.085**	**0.311**	**0.605**	**0.830**	**0.930**	**0.948**
	3	*WL*	0.075	0.253	0.555	0.786	0.911	0.931
		*FQLS*_1_	**0.079**	0.298	0.592	0.813	0.921	0.972
		*FQLS*_2_	0.078	**0.311**	**0.629**	**0.823**	**0.941**	**0.982**
	4	*WL*	0.081	0.307	0.585	0.800	0.917	0.951
		*FQLS*_1_	0.088	**0.325**	**0.622**	0.828	0.928	0.957
		*FQLS*_2_	**0.092**	0.318	0.614	**0.832**	**0.929**	**0.962**
0.001	1	*WL*	0.016	0.074	0.229	0.444	0.602	0.710
		*FQLS*_1_	0.017	0.072	0.221	0.436	0.643	0.693
		*FQLS*_2_	**0.021**	**0.120**	**0.309**	**0.573**	**0.739**	**0.820**
	2	*WL*	0.020	0.074	0.251	0.436	0.676	0.778
		*FQLS*_1_	0.017	0.103	0.313	0.540	0740	0.798
		*FQLS*_2_	**0.024**	**0.116**	**0.350**	**0.589**	**0.782**	**0.845**
	3	*WL*	0.024	0.081	0.278	0.513	0.734	0.810
		*FQLS*_1_	0.016	0.124	0.341	0.581	0.769	0.864
		*FQLS*_2_	**0.017**	**0.129**	**0.365**	**0.604**	**0.802**	**0.881**
	4	*WL*	0.015	0.109	0.310	0.542	0.756	0.830
		*FQLS*_1_	**0.021**	0.118	0.335	0.588	0.783	0.867
		*FQLS*_2_	**0.021**	**0.133**	**0.345**	**0.597**	**0.790**	**0.874**

The bold text indicates the highest empirical estimate of the power for each situation

The bold text indicates the highest empirical estimate of the power for each situation

**Table 5:** Empirical power estimates for scenario 2 when *h* ^2^ is 0.2. The empirical power estimates for scenario 2 were calculated with 1000 replicates at the both 0.01, and 0.001 significance levels. The disease allele frequency was assumed to be 0.2, and the prevalence was assumed to 0.2. The relative phenotypic variance attributable to the main disease gene was assumed to be 0.005

	*n*_*proband*_	Statistic	*N*			
			100	300	600	900
0.01	1	*WL*	0.072	0.149	0.304	0.409
		*FQLS*_1_	0.072	0.147	0.305	0.415
		*FQLS*_2_	**0.075**	**0.158**	**0.312**	**0.434**
	2	*WL*	0.042	0.137	0.300	0.448
		*FQLS*_1_	0.039	0.136	**0.303**	0.455
		*FQLS*_2_	**0.041**	**0.139**	0.295	**0.471**
	3	*WL*	**0.058**	0.188	0.410	0.608
		*FQLS*_1_	0.054	**0.191**	**0.424**	**0.620**
		*FQLS*_2_	0.055	0.190	0.423	0.615
0.001	1	*WL*	**0.025**	0.059	0.147	0.211
		*FQLS*_1_	0.023	**0.062**	**0.152**	0.197
		*FQLS*_2_	0.022	0.055	**0.152**	**0.212**
	2	*WL*	0.010	**0.038**	0.123	0.229
		*FQLS*_1_	**0.012**	0.036	0.123	**0.232**
		*FQLS*_2_	0.010	0.036	**0.127**	0.227
	3	*WL*	0.006	0.055	**0.182**	0.342
		*FQLS*_1_	0.007	0.055	**0.182**	0.355
		*FQLS*_2_	**0.009**	**0.057**	**0.182**	**0.356**

The bold text indicates the highest empirical estimate of the power for each situation

The bold text indicates the highest empirical estimate of the power for each situation

**Table 6:** Empirical power estimates for scenario 2 when *h* ^2^ is 0.5. The empirical power estimates for scenario 2 were calculated with 1000 replicates at the both 0.1 and 0.001 significance levels. The disease allele frequency was assumed to be 0.2, and the prevalence was assumed to 0.2. The relative phenotypic variance attributable to the main disease gene was assumed to be 0.005

	*n*_*proband*_	Statistic	*N*			
			100	300	600	900
0.01	1	*WL*	0.130	0.293	0.567	0.787
		*FQLS*_1_	0.130	0.295	0.568	0.773
		*FQLS*_2_	**0.140**	**0.323**	**0.603**	**0.823**
	2	*WL*	0.093	0.332	0.645	0.864
		*FQLS*_1_	0.094	**0.357**	0.654	0.871
		*FQLS*_2_	**0.108**	0.354	**0.694**	**0.902**
	3	*WL*	0.100	0.382	0.735	0.904
		*FQLS*_1_	0.108	0.406	0.751	0.915
		*FQLS*_2_	**0.130**	**0.408**	**0.772**	**0.932**
0.001	1	*WL*	0.046	0.148	0.341	0.560
		*FQLS*_1_	**0.050**	0.149	0.353	0.559
		*FQLS*_2_	0.047	**0.166**	**0.386**	**0.617**
	2	*WL*	0.019	0.127	0.387	0.634
		*FQLS*_1_	**0.021**	0.119	0.394	0.648
		*FQLS*_2_	0.017	**0.144**	**0.432**	**0.695**
	3	*WL*	0.023	0.166	0.481	0.749
		*FQLS*_1_	0.026	0.183	0.511	0.772
		*FQLS*_2_	**0.028**	**0.196**	**0.532**	**0.782**

The bold text indicates the highest empirical estimate of the power for each situation

The bold text indicates the highest empirical estimate of the power for each situation

**Table 7:** Empirical power estimates for scenario 2 when *h* ^2^ is 0.8. The empirical power estimates for scenario 2 were calculated with 1000 replicates at the both 0.01 and 0.001 significance levels. The disease allele frequency was assumed to be 0.2, and the prevalence was assumed to 0.2. The relative phenotypic variance attributable to the main disease gene was assumed to be 0.005

	*n*_*proband*_	Statistic	*N*			
			100	300	600	900
0.01	1	*WL*	0.164	0.445	0.749	0.906
		*FQLS*_1_	0.156	0.441	0.751	0.905
		*FQLS*_2_	**0.194**	**0.508**	**0.817**	**0.944**
	2	*WL*	0.132	0.473	0.823	0.970
		*FQLS*_1_	0.131	0.505	0.861	0.969
		*FQLS*_2_	**0.150**	**0.564**	**0.905**	**0.978**
	3	*WL*	0.140	0.475	0.835	0.958
		*FQLS*_1_	0.134	0.520	0.867	0.970
		*FQLS*_2_	**0.167**	**0.556**	**0.886**	**0.981**
0.001	1	*WL*	0.059	0.230	0.519	0.759
		*FQLS*_1_	0.053	0.236	0.519	0.757
		*FQLS*_2_	**0.070**	**0.311**	**0.632**	**0.841**
	2	*WL*	0.039	0.239	0.561	0.858
		*FQLS*_1_	0.033	0.250	0.594	0.884
		*FQLS*_2_	**0.053**	**0.300**	**0.702**	**0.924**
	3	*WL*	0.033	0.215	0.629	0.865
		*FQLS*_1_	**0.046**	0.247	0.671	0.900
		*FQLS*_2_	0.044	**0.287**	**0.713**	**0.925**

The bold text indicates the highest empirical estimate of the power for each situation

The bold text indicates the highest empirical estimate of the power for each situation

### Robustness of the proposed methods against the misspecification of prevalence

The statistical powers of the proposed methods may depend on the accuracy of the prevalence and we evaluated the sensitivity of the proposed method to the misspecified prevalence with simulated data. *h*_*a*_^2^ and *h*^2^ were assumed to be 0.05 and 0.8, and nuclear families (Fig. 2-(a)) were considered in this simulation. The number of nuclear families was assumed to be 900 and *n*_*proband*_ was assumed to be 1, 2, or 3. Prevalence was assumed to be 0.2 for phenotype generation, and the offset for which we recommended prevalence was set to be 0.1, 0.2 or 0.3 for calculation of *FQLS*_1_ and *FQLS*_2_. In particular, *y*_*ij*_ for individuals with missing phenotypes are coded by the assumed prevalence, and sensitivity of the proposed methods can be substantial when there are individuals with missing phenotypes. Therefore, individuals were randomly selected from non-probands, and their phenotypes were assumed to be unknown for calculation of the proposed statistics. The number of family members with missing phenotypes in each family was denoted by *n*_*missing*_. The empirical powers were calculated at the 0.01 significance level with 1000 replicates. Table 8 shows that the results obtained by setting prevalence to be 0.1 and 0.3 are similar to the results when the prevalence was set to be 0.2, which indicates that the power loss attributable to the misspecified prevalence is not substantial. Furthermore the empirical power estimates are positively related with *n*_*proband*_ and inversely related with *n*_*missing*_. If *n*_*missing*_ is larger than 3, the power loss may be more substantial.

**Table 8:** Empirical power estimates for three situations when *h* ^2^ is 0.8. The empirical power estimates for three situations were calculated with 1000 replicates at the 0.01 significance levels. Phenotypes were generated under the assumption that the prevalence was assumed to 0.2. Prevalence was set to be 0.1, 0.2 or 0.3 to calculate the proposed statistics. The relative phenotypic variance attributable to the main disease gene was assumed to be 0.005

*n*_*missing*_	*n*_*proband*_	Statistic	Prevalence to be set for statistics		
			0.1	0.2	0.3
0	1	*FQLS*_1_	0.486	**0.479**	**0.464**
		*FQLS*_2_	**0.572**	**0.539**	0.525
	2	*FQLS*_1_	0.562	**0.557**	0.532
		*FQLS*_2_	**0.606**	**0.621**	0.624
	3	*FQLS*_1_	0.632	**0.642**	0.634
		*FQLS*_2_	0.644	**0.647**	0.654
1	1	*FQLS*_1_	0.432	**0.434**	**0.418**
		*FQLS*_2_	0.468	**0.469**	0.462
	2	*FQLS*_1_	0.522	**0.530**	0.519
		*FQLS*_2_	0.547	**0.554**	0.559
	3	*FQLS*_1_	0.580	**0.584**	0.576
		*FQLS*_2_	0.575	**0.584**	0.585
2	1	*FQLS*_1_	0.415	**0.412**	0.399
		*FQLS*_2_	0.428	**0.420**	0.412
	2	*FQLS*_1_	0.482	**0.487**	**0.468**
		*FQLS*_2_	0.483	**0.495**	0.492
	3	*FQLS*_1_	0.487	**0.497**	0.490
		*FQLS*_2_	**0.500**	**0.515**	0.511

The bold text indicates the reference for the proposed statistics to compare with the misspecified prevalence

The bold text indicates the reference for the proposed statistics to compare with the misspecified prevalence.

### Application of the proposed method to AD

AD is an irreversible, progressive brain disorder characterized by genetic heterogeneity. However, the genetic variations that contribute to AD still remain elusive. Thus, we applied the proposed method for identification of the disease susceptibility loci for AD. The heritability and prevalence of AD are approximately 0.8 [29, 30] and 0.1, respectively; therefore, we chose heritabilities of 0.8, and a prevalence of 0.1 for the calculation of proposed methods. Samples were collected as part of the National Institute of Mental Health Genetics Initiative (NIMH). The NIMH Alzheimer’s Disease Genetics Family Sample was used along with the information about the genotype platform (Affy 6.0) [20, 31], and ethical approach and participant approval were obtained through the NIMH IRB panel. Families were selected based on the disease status of a certain family member. However the proband for each family was not clear, and *FQLS*_2_ was uniquely applied for the proposed method. 1376 individuals from 410 families were available, and all families were nuclear. All individuals were of self-reported European ancestry. HWE for each single nucleotide polymorphism (SNP) was tested, and MAFs were estimated. SNPs for which *p*-values for HWE were less than 10^−6^ or MAFs less than 0.05 were excluded, and therefore, 417,680 SNPs were analyzed for genetic association analysis.

**Fig. 4:**
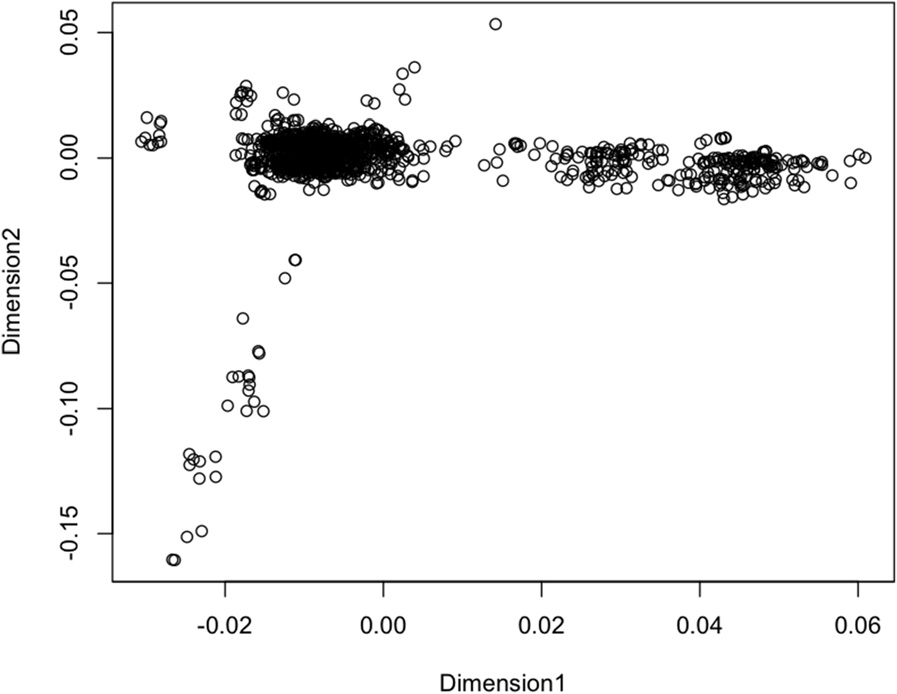
Multidimensional scaling plots from samples for the GWAS for AD. Founders were selectively used, and multidimensional scaling plots were obtained with the first and second PC scores

**Fig. 5:**
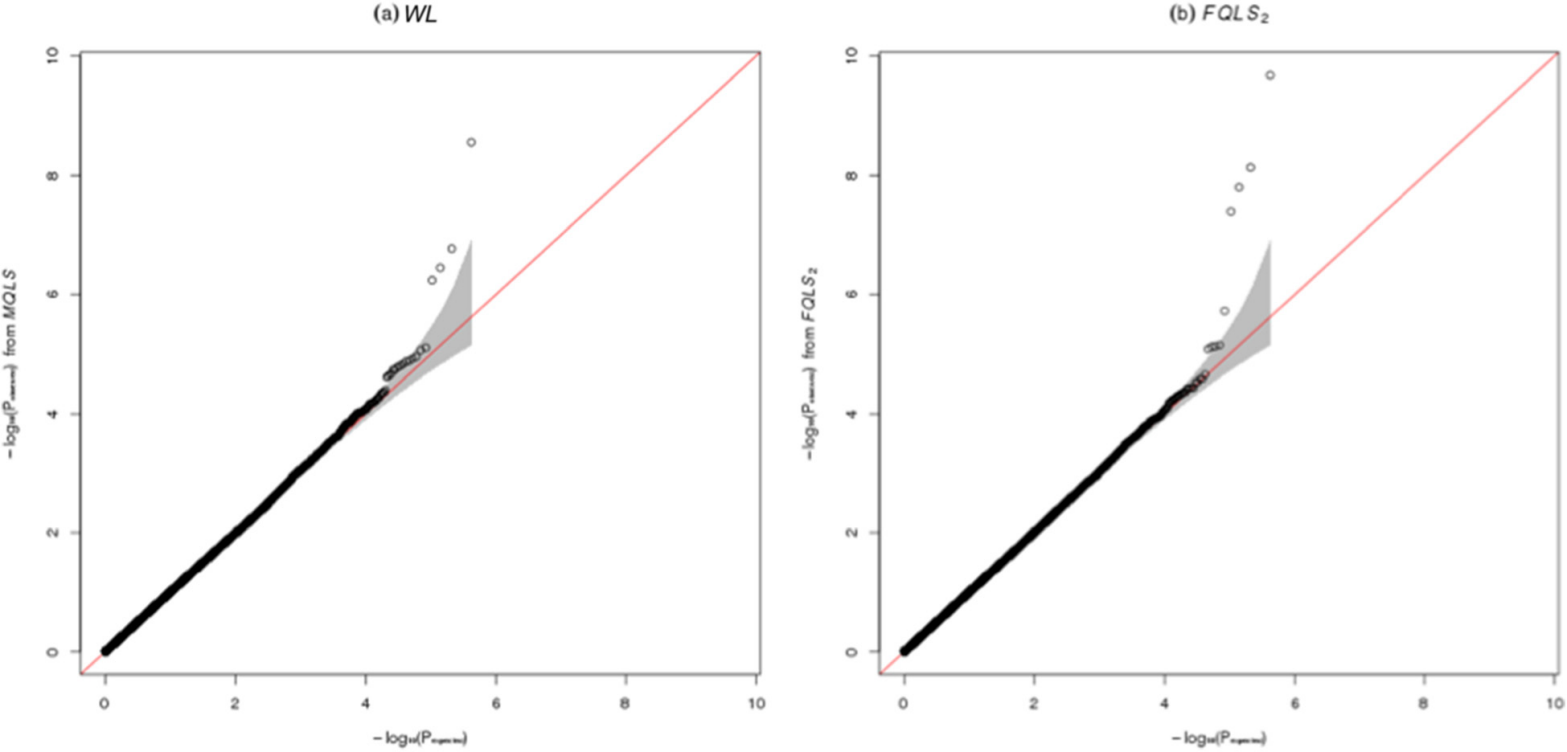
QQ plots of results from GWAS for AD. QQ plots are provided with the results from **(a)** *WL* and **(b)** *FQLS*_2_

The choice of kinship coefficient matrix for the proposed method depends on the presence of population substructure. To confirm the presence of population substructure, multidimensional-scaling [32] analysis was performed with PLINK1.07 [20], and we constructed a multidimensional-scaling plot (Fig. 4) to provide evidence concerning the presence of population substructure. Therefore, the genomic control method [31, 33, 34] was used for explicit detection and correction of population stratification with common SNPs, and they were incorporated into both *FQLS*_2_ and *WL*. Fig. 5 shows QQ plots for *FQLS*_2_ and *WL*; these plots revealed that the presence of population substructure was appropriately adjusted. Results showed four genome-wide significant results for *FQLS*_2_ and one significant result for *WL*.

Detailed information for these significant results is provided in Table 9. We also considered FBAT statistics [35]. *FQLS*_2_ identified four genome-wide significant SNPs, while *WL* and FBAT identified one genome-wide significant SNP. In addition, one SNP that was significant according to *WL* was more significant according to *FQLS*_2_. The most significant result acquired using *FQLS*_2_ was SNP4 (*p* = 2.10× 10^−10^). The other three SNPs, i.e., SNP1, SNP2 and SNP3, reached the genome-wide significance level.

**Table 9:** Top significant results of GWAS for AD. For the genome-wide significant SNPs from *FQLS*_2_ and *WL*, their *p*-values are given

SNP	*WL*	*FQLS*_2_	*FBAT*
SNP1	3.53× 10^−7^	1.94× 10^−8^	4.23× 10^−4^
SNP2	5.74× 10^−7^	4.45× 10^−8^	4.9× 10^−5^
SNP3	1.69× 10^−7^	8.36× 10^−9^	8.6× 10^−5^
SNP4	2.79× 10^−9^	2.86× 10^−10^	6.94× 10^−12^

## Discussion

Although major advances in high-density genome scans have enabled the genetic association analysis of more than 10,000 individuals, disappointing results in the mapping of many common diseases have illustrated the need for more powerful methods for detecting disease susceptibility loci. Statistical efficiency is known to being affected by the ascertainment bias, and its careful adjustment can lead to substantial improvement of statistical power [36–38]. In particular, genetic association analysis has often been conducted using family-based designs, but without addressing the fact that the probability for each family member to be affected is inversely related with the familial relationship with affected probands. In this report, we proposed new methods to adjust this heterogeneity with known prevalence and heritability. Our simulation studies showed that the proposed methods provided substantial power improvement. In particular, the mis-specified heritability and prevalence can lead to the statistical power loss for the proposed methods, but it was found to be not substantial at least in our simulation studies. *FQLS*_1_ and *FQLS*_2_ were suggested, and *FQLS*_1_ is an efficient choice if probands for each family are clearly defined and all remaining family members are incorporated to the genetic analysis. However, these conditions are often not satisfied, and different methods such as sequential sampling frame [28] are usually utilized. Simulation studies showed that *FQLS*_2_ is usually better than *FQLS*_1_ if the ascertaining condition is not clearly defined and thus we recommend *FQLS*_2_ unless probands are clearly defined. However we considered the limited ascertainment conditions and comprehensive simulation studies are still necessary.

Furthermore, the proposed method was conceptually simple and can be applied to the large families. Our methods require only a single calculation of offset for all markers, and the real data analysis could be completed with a single CPU in a few hours. For *M* markers and *N* individuals, the time complexity is *O*(*N*^3^ + *MN*^2^) for the proposed method. The proposed method was implemented with C++, and can be downloaded from http://healthstat.snu.ac.kr/mfqls/.

Heterogeneity between samples is an important issue in large-scale genetic analysis, and the proposed method can likely be applied to various additional scenarios with some modifications. For instance, the disease status of relatives reveals the importance of genetic components for each individual, and for this reason, such information has been used, albeit only on occasion, in genetic association analysis. The effect of relatives’ disease statuses is dependent on prevalence and heritability, and the probability for each individual to be affected could be calculated with the proposed method. This probability can be used to improve the statistical efficiency of genetic association analysis. In addition, the heterogeneity of the ascertainment bias is often an important issue for genome-wide meta-analysis because samples are collected from multiple medical centers [39, 40], and different sampling schemes among studies need to be adjusted to improve statistical efficiency. Therefore, we believe that proposed method can be extended to provide a statistical framework that adjusts the heterogeneity between samples.

## Conclusions

We proposed *FQLS* method to adjust this heterogeneity with known prevalence and heritability and the software was implemented with C++. We identified several significant associations between AD and SNPs, and their potential functional information will provide the better understanding of the pathogenesis of AD. Although this study has some limitations, our proposed methods illustrated important features required for genetic analysis with family-based samples, and an extension of the proposed method to rare variant association analysis such as *FARVAT* [41] will be investigated in future studies.

## Additional file

Additional file 1:
**Web-based Supporting Materials for “Adjusting heterogeneous ascertainment bias for genetic association analysis with extended families” by Suyeon Park, Sungyoung Lee, Young Lee, Christine Herold , Basavaraj Hooli, Kristina Mullin, Lars Bertram, Taesung Park, Changsoon Park, Christoph Lange, Rudolph Tanzi , and Sungho Won.**

## Abbreviations

GWAS: Genome-wide association studies
CA: Cochran-Armitage
AD: Alzheimer’s diseas
PD: Parkinson’s disease
HWE: Hardy-Weinberg equilibrium
BLUE: Best linear unbiased estimator
QQ: Quantile quantile
MVN: Multivariate normal
MAF: Minor allele frequency
SNP: Single nucleotide polymorphism
FBAT: Family Based Association Testing software
FQLS: Family based quasi-likelihood score test
