# Supplementary material for: Adjusting heterogeneous ascertainment bias for genetic association analysis with extended families
Source: BMC Med Genet. 2015 Aug 19;16:62. doi: 10.1186/s12881-015-0198-6 (PMC4593209; doi:10.1186/s12881-015-0198-6)
Supplement: Additional file 1: — Web-based Supporting Materials for “Adjusting heterogeneous ascertainment bias for genetic association analysis with extended families” by Suyeon Park, Sungyoung Lee, Young Lee, Christine Herold , Basavaraj Hooli, Kristina Mullin, Lars Bertram, Taesung Park, Changsoon Park, Christoph Lange, Rudolph Tanzi , and Sungho Won. [file 12881_2015_198_MOESM1_ESM.docx]

**Web-based Supporting Materials for “Adjusting heterogeneous ascertainment bias for genetic association analysis with extended families” by Suyeon Park, Sungyoung Lee, Young Lee, Christine Herold , Basavaraj Hooli, Kristina Mullin, Lars Bertram, Taesung Park, Changsoon Park, Christoph Lange, Rudolph Tanzi , and Sungho Won**

The optimal choices of offset can be identified by minimizing the variance and if we let **V** = **I***_N_* and

$$\mathbf{\Sigma}=\sigma^{2}\left(\mathbf{R}-\mathbf{1}_{N}\left(\mathbf{1}_{N}^{t}\mathbf{R}^{-1}\mathbf{1}_{N}\right)^{-1}\mathbf{1}_{N}^{t}\right),$$

the denominator of the non-centrality parameter became

$$\mathrm{var}(S)=\text{tr}\left\{\mathbf{\Sigma TT}^{t}\right\}.$$

However the amount of variance depends on the realization of **Y**, and there is no globally optimal choice of offset. Alternatively we focus on expected variance of S:

$$E\left\{\mathrm{var}(S)\right\}=E\left[\text{tr}\left(\mathbf{\Sigma TT}^{t}\right)\right]=\text{tr}\left[\mathbf{\Sigma}E\left(\mathbf{TT}^{t}\right)\right].$$

We assumed that the dichotomous phenotype was conditioned, and MAFs were compared between affected and unaffected individuals. For ascertained families, the probability for each individual to be affected could be related to the phenotypes of other family members, and we let **Y**-(*_ij_*) be the phenotype vector where the phenotype of individual *j* in family *i* was excluded. We denoted the offset for individual *j* in family *i* by *μ_ij_*. If we let

$\mu_{ij}^{*}=E(y_{ij}|\mathbf{Y}_{-(ij)}),\text{ }\mathbf{\mu}_{i}^{*}=\left(\begin{matrix} \mu_{i1}^{*} \\ \vdots\\ \mu_{in_{i}}^{*} \end{matrix}\right),\text{ }\mathbf{\mu}_{i}=\left(\begin{matrix} \mu_{i1} \\ \vdots\\ \mu_{in_{i}} \end{matrix}\right),\text{ }\mathbf{\mu}^{*}=\left(\begin{matrix} \mathbf{\mu}_{1}^{*} \\ \vdots\\ \mathbf{\mu}_{n}^{*} \end{matrix}\right),\text{ and }\mathbf{\mu}=\left(\begin{matrix} \mathbf{\mu}_{1} \\ \vdots\\ \mathbf{\mu}_{n} \end{matrix}\right)$,

we obtained

$$\begin{matrix} \mathbf{TT}^{t}=\left(\mathbf{Y}-\mathbf{\mu}^{*}+\mathbf{\mu}^{*}-\mathbf{\mu}\right)\left(\mathbf{Y}-\mathbf{\mu}^{*}+\mathbf{\mu}^{*}-\mathbf{\mu}\right)^{t} \\ \text{ }=\left(\mathbf{Y}-\mathbf{\mu}^{*}\right)\left(\mathbf{Y}-\mathbf{\mu}^{*}\right)^{t}+\left(\mathbf{\mu}^{*}-\mathbf{\mu}\right)\left(\mathbf{\mu}^{*}-\mathbf{\mu}\right)^{t}-2\left(\mathbf{Y}-\mathbf{\mu}^{*}\right)\left(\mathbf{\mu}^{*}-\mathbf{\mu}\right)^{t}. \end{matrix}$$

In particular, the certain constant such as prevalence was often utilized for *μ_ij_* and if we let the chosen offset be *μ*,

$$\begin{matrix} E\left\{\left(y_{ij}-\mu_{ij}^{*}\right)\left(\mu_{ij}^{*}-\mu\right)^{t}\right\} \\ \text{ }=E\left[E\left(\left.\left(y_{ij}-E(y_{ij}|\mathbf{Y}_{-(ij)})\right)\left(E(y_{ij}|\mathbf{Y}_{-(ij)})-\mu\right)^{t}\right|\mathbf{Y}_{-(ij)}\right)\right] \\ \text{ }=E\left[\left\{E\left(\left.y_{ij}-E(y_{ij}|\mathbf{Y}_{-(ij)})\right|\mathbf{Y}_{-(ij)}\right)\right\}\left(E(y_{ij}|\mathbf{Y}_{-(ij)})-\mu\right)^{t}\right]=0. \end{matrix}$$

Therefore, we obtained the following inequality for the denominator of the non-centrality parameter:

$$E\left[\text{tr}\left\{\mathbf{\Sigma TT}^{t}\right\}\right]\geq E\left[\text{tr}\left\{\left(\mathbf{Y}-\mathbf{\mu}^{*}\right)^{t}\mathbf{\Sigma}\left(\mathbf{Y}-\mathbf{\mu}^{*}\right)+\left(\mathbf{\mu}^{*}-\mathbf{\mu}\right)^{t}\mathbf{\Sigma}\left(\mathbf{\mu}^{*}-\mathbf{\mu}\right)\right\}\right],$$

and the equality was satisfied at $\mu_{ij}=\mu_{ij}^{*}$.
